# Association of duration of treatment on post-discharge mortality in forensic psychiatric patients in Finland

**DOI:** 10.3389/fpsyt.2024.1372687

**Published:** 2024-08-19

**Authors:** Ilkka Ojansuu, Jonas Forsman, Hannu Kautiainen, Allan Seppänen, Jari Tiihonen, Markku Lähteenvuo

**Affiliations:** ^1^ Department of Forensic Psychiatry, Niuvanniemi Hospital, University of Eastern Finland, Kuopio, Finland; ^2^ Department of Clinical Neuroscience, Karolinska Institutet, Stockholm, Sweden; ^3^ The Swedish National Board of Forensic Medicine, Department for Forensic Psychiatry, Stockholm, Sweden; ^4^ Primary Health Care Unit, Kuopio University Hospital, Kuopio, Finland; ^5^ Folkhälsan Research Center, Helsinki, Finland; ^6^ Centre for Psychiatry Research, Department of Clinical Neuroscience, Karolinska Institutet, Stockholm, Sweden; ^7^ Stockholm Health Care Services, Region Stockholm, Stockholm, Sweden

**Keywords:** psychosis, schizophrenia, substance use disorder, mortality, treatment time

## Abstract

**Background:**

Longer treatment time has been shown to be associated with lower crime recidivism among forensic psychiatric patients, but it is not known if this applies also to mortality. In this study, we aim to research whether treatment time is associated with risk of post-discharge mortality in Finnish forensic psychiatric patients.

**Materials and methods:**

The study population consisted of 989 patients committed to compulsory forensic psychiatric hospital treatment in Finland from 1980 to 2009 who were released from care by the end of 2018. Each patient included in the cohort was linked with the Statistics Finland register, which includes all data on dates and causes of deaths in Finland. Crude cumulative rate of mortality were estimated using Kaplan–Meier method and compared using logrank-test. Adjusted cumulative rate analyzed using Cox regression model. A possible nonlinear relationship between the treatment time and the hazard of death was assessed by using 3-knot-restricted cubic spline regression. Adjusted models included age, sex, and SUD (substance use disorder) as covariates.

**Results:**

The mean duration of care was 7.1 (SD 6) years. The duration of treatment variable was divided into tertiles of treatment duration less than 3.5 years, 3.5–7.9 years and equal or more than 8 years. The risk of mortality was highest in the first tertile, and lowest in the last tertile. The risk of mortality was higher for patients suffering from SUD, for patients of male sex and for those released at younger age.

**Conclusions:**

Longer treatment time is associated with reduced post-discharge mortality in forensic psychiatric patients in Finland. Especially males and individuals with SUD are at higher mortality risk after release, but longer treatment duration may mitigate these risks. Longer periods of hospitalization have to be, however, viewed against the backdrop of institutionalization and loss of self-determination.

## Introduction

It has been known for decades that psychotic disorders are associated with increased mortality (1). In schizophrenia, mortality has been shown to be elevated both due to somatic diseases as well as unnatural causes, such as suicide, accidents and violence (2–4). Moreover, substance use disorders (SUDs) have been shown to be prevalent in psychotic disorders, and several studies show that they increase the mortality of psychiatric patients (5–7).

In a recent meta-analysis of 135 studies the relative risk of all‐cause mortality was increased (RR 2.52, 95% CI: 2.38‐2.68) in people with schizophrenia versus the general population (8). Relative risk of cause-specific mortality was highest for suicide, injury‐poisoning or undetermined non‐natural cause (RR 9.76‐8.42). Comorbid substance use disorder increased all‐cause mortality (RR 1.62, 95% CI: 1.47‐1.80) and antipsychotics use was protective against all‐cause mortality (RR 0.71, 95% CI: 0.59‐0.84). Results of the meta-analysis indicated that the excess mortality in schizophrenia is associated with several modifiable factors.

In Finland, the forensic patient population consists of individuals who have committed a crime and, instead of being sentenced to prison, have been committed due to a psychotic disorder to involuntary forensic psychiatric treatment by the Finnish Institute for Health and Welfare (THL). Treatment times in forensic care are often markedly longer than in general psychiatric care. The patients often have comorbidities such as SUDs and personality disorders, but these disorders in themselves, without a psychotic disorder, are not considered to be ground for forensic care. As comorbidities in patients are frequent, they have cumulated risk factors for increased mortality, since also criminal activity and antisociality has been associated with increased mortality (9, 10).

In a previous study including 950 patients in Finland who were released from forensic care with a mean 13.4-year follow-up, we have shown that the standardized mortality ratio (SMR) of forensic patients was 3.5 times that of the general population (11). The SMR was higher for those with a comorbid SUD at the beginning of treatment (SMR 4.1) than for those without such a comorbidity (SMR 2.8). The results were similar to results of the mortality among forensic psychiatric patients in Japan (SMR 2.2) and in England (SMR 6.3) (12, 13).

In a Swedish study with a mixed population of 6520 patients in both forensic and general psychiatric care after court decision, who were released from care between the years 1973–2009, 1949 (30%) patients died during a mean follow-up of 15.6 years (14). The patients had been treated for an average treatment time of 5.1 months (Interquartile range: 1.7–12.7 months). In this study, a longer duration of treatment was associated with reduced mortality in general, but not after adjusting for relevant risk factors (age at discharge, sex, previous violent offence, index violent offence, primary diagnosis, secondary substance use disorder and secondary personality disorder).

In a Danish study the mortality of 490 male forensic psychiatric patients, who were committed to forensic psychiatric treatment during the years 1980–1992, was compared to the mortality of 490 age matched psychiatric male patients and 1716 males in the general population as controls (15). Out of the forensic patients, 63% had a major psychiatric disorder, 19% a personality disorder and 18% another disorder as primary diagnosis. Mean follow-up time in the study was 19 years, under which 213 (44%) of the forensic psychiatric patients died. The Danes found that in their clinical cohorts of both forensic and non-forensic psychiatric patients (of whom the majority suffered from a psychotic disorder), a longer admission time was associated with higher mortality. However, the authors speculated that longer length of admission may have been a proxy for a higher severity of disorder, including severe comorbidities, or higher prevalence of medication non-adherence. In this study also the lifetime diagnosis of SUD was associated with higher mortality.

Comparing mortality in forensic psychiatric patients between countries is however problematic, since countries differ in their criteria of what disorders mandate forensic psychiatric care, for example whether SUDs or personality disorders in themselves mandate care. Such differences in patient populations may naturally also affect the content of treatment as well as prognosis. There are also different legal frameworks for inpatient and outpatient treatment as well as for rehospitalization.

Evidently the previous literature on effects of treatment time on mortality among forensic psychiatric patients is scarce. Longer duration of treatment has been shown to reduce other negative outcomes, such as criminal recidivism, which in itself has been linked to risk of mortality. Thus, the relationship of treatment time and mortality warrants further study, especially as mortality is regarded as the most robust outcome measure of illnesses and an important standard for measuring clinical performance (16). It is also an important indicator in psychiatric care where one of the ways practices and services are assessed is to see how well they reduce mortality. The Finnish system of recording causes of death, which has been found to be reliable and extensive, provides a solid foundation for studying mortality in Finland (17).

The aim of the study is to provide more information on the relationship between duration of treatment and mortality in forensic psychiatric patients with a psychotic disorder. This information is crucial when developing the practices of forensic care in order to both reduce excess mortality, but also to keep in-hospital treatment-times to the minimum, reduce the extent to which the rights to freedom of the patients are affected, as well as the economic costs to society.

## Materials and methods

### Cohort and data collection

The study population consisted of patients committed to compulsory forensic psychiatric hospital treatment in Finland from 1980 to 2009 who were released from care by the end of 2018. The study population was collected from the archive of the National Institute for Health and Welfare (THL).

The mental state of all patients included in the data was examined by THL in accordance with a court order. The patients had been diagnosed with a psychotic disorder during their forensic psychiatric examination and had been found criminally irresponsible for the crime for which they were ordered to attend a forensic psychiatric examination. The patients had been committed to involuntary forensic psychiatric treatment instead of being sentenced to prison.

The forensic psychiatric examination notes were used to record substance use disorder comorbidities. The examinations were also reviewed by one of the authors (Dr. Ilkka Ojansuu) in order to identify any SUDs that were described in the statement, but for which no diagnosis was given. Any patient with evidence of substance dependence syndrome or harmful use (ICD-10: F1x.1–F1x.2) was counted as having an SUD regardless of the substance. Based on the collected data, the patients were divided into two groups depending on whether they were suffering from SUD or not at the time of the forensic psychiatric examination. If there was evidence in the forensic psychiatric examination statement of only intoxication or withdrawal symptoms without a longer standing substance abuse disorder or only prior evidence of SUDs without current use, the patient was included to the group of non-SUD among those patients without any evidence of an SUD.

The treatment time consisted of all compulsory care, which included both in-hospital treatment as well as a possible supervision period of compulsory out-of-hospital treatment. Not all patients had this supervised outpatient care, which in Finland can last for up to 6 months at a time (although there can be multiple periods) and during which time the forensic patients are still legally considered to be in in-hospital care and can be readmitted to hospital care if needed. The follow-up of patients began after release from treatment i.e. after both hospital treatment and possibly followed supervision period had been terminated and lasted until the end of January 2020. Release from compulsory care in Finland is determined by the mental health act (“Mielenterveyslaki”) and has to occur when compulsory care is no longer necessary, e.g. at the point where the patient’s psychiatric condition is deemed to be stabilized and the risk for reoffending is assessed as being sufficiently low) (18). After the forensic psychiatric care ended, there is no mandatory outpatient treatment, although most patients are advised to attend voluntary outpatient care. The Finnish Forensic Psychiatric system has been detailed in a previous publication (18).

In order to analyze the mortality data, the personal identity code of each patient included in the cohort was linked with the Statistics Finland register, which includes all data on dates and causes of deaths in Finland.

### Statistical analyses

The data are expressed as mean and standard deviation (SD), or count and percentage (%), as appropriate. Crude cumulative rate of mortality were estimated using Kaplan– Meier method and compared between groups with the logrank test. Adjusted cumulative rate analyzed using Cox regression model. A possible nonlinear relationship between the treatment time and the hazard of death was assessed by using 3-knot-restricted cubic spline regression. Adjusted models included age, sex, and SUD as covariates. Stata 17.0 (StataCorp LP; College Station, Texas, USA) statistical package was used for the analysis.

### Ethical considerations

This study was purely register based and no contacts were made with the subjects of the study. The study was approved by THL and by Statistics Finland. The ethical review for the project was conducted by the Finnish Institute for Health and Welfare prior to granting access to the registry data. All data were analyzed in pseudonymized form.

## Results

During the follow-up period, out of the total of 1253 patients committed to care, the THL Board for Forensic Psychiatric Affairs ended the forensic psychiatric treatment for 989 patients (857 men, 132 women). The mean age of the patients at the start of treatment was 37 years (SD 13) among both males and females, and 44 (SD 13) years at discharge from care (44 for men and 43 for women). At the time of discharge the mean duration of care was 7.1 (SD 6) years, (7.3 (SD 6) for males and 5.9 (SD 5) for females. The mean follow-up time after discharge from care was 21.7 (SD 9.3) years, 21.8 (SD 9.1) for women and 21.7 (SD 9.3) for men. The follow-up time accumulated a total of 21 504 person-years (18 628 for men and 2 876 for women.)

All patients included in the data had a psychotic disorder, mostly (87%) in the schizophrenia spectrum (ICD-10: F20–29). Of these, 68% had schizophrenia (F20.x), 13% had a delusional disorder (F22.x) and 9% had a schizoaffective disorder (F25.x). Out of the 989 patients discharged from care, 598 (60.5%) had a comorbid substance use disorder according to ICD-10 criteria of either dependence or harmful use in conjunction with their psychotic disorder during their forensic psychiatric examination. Of these, 540 (90.3%) were men and 58 (9.7%) women.

Of the 989 discharged, 416 (372 men, 44 women) died during the follow-up period. The demographics of the total cohort and the individuals who died during follow-up are shown in **Table 1**. The mean age at time of death was 59.9 years (59.8 for men, 60.9 for women). The cause of death was unnatural for 90 patients (83 male, 7 female) of which there were 39 suicides (35 male, 4 female), 48 accidental deaths (45 male, 3 female) and 3 homicides (3 male, 0 female). The cause of death was natural for 315 patients (279 male, 36 female). For 11 patients (10 male, 1 female) the cause of death remained unclear.

**Table 1:** Demographics of the total cohort and the individuals who died during follow-up.

	Total cohort	Individual who died during follow-up
Mean age at discharge (years)	44 (SD 13)	49 (SD 14)
Males (n, %)	857 (86.7%)	372 (89.4%)
Substance use disorder	598 (60.5%)	262 (63.0%)
Mean treatment time (years)	7.1 (SD 5.9)	6.0 (SD 5.4)
Diagnosis F20–29	87%	84%

**Figure 1:**
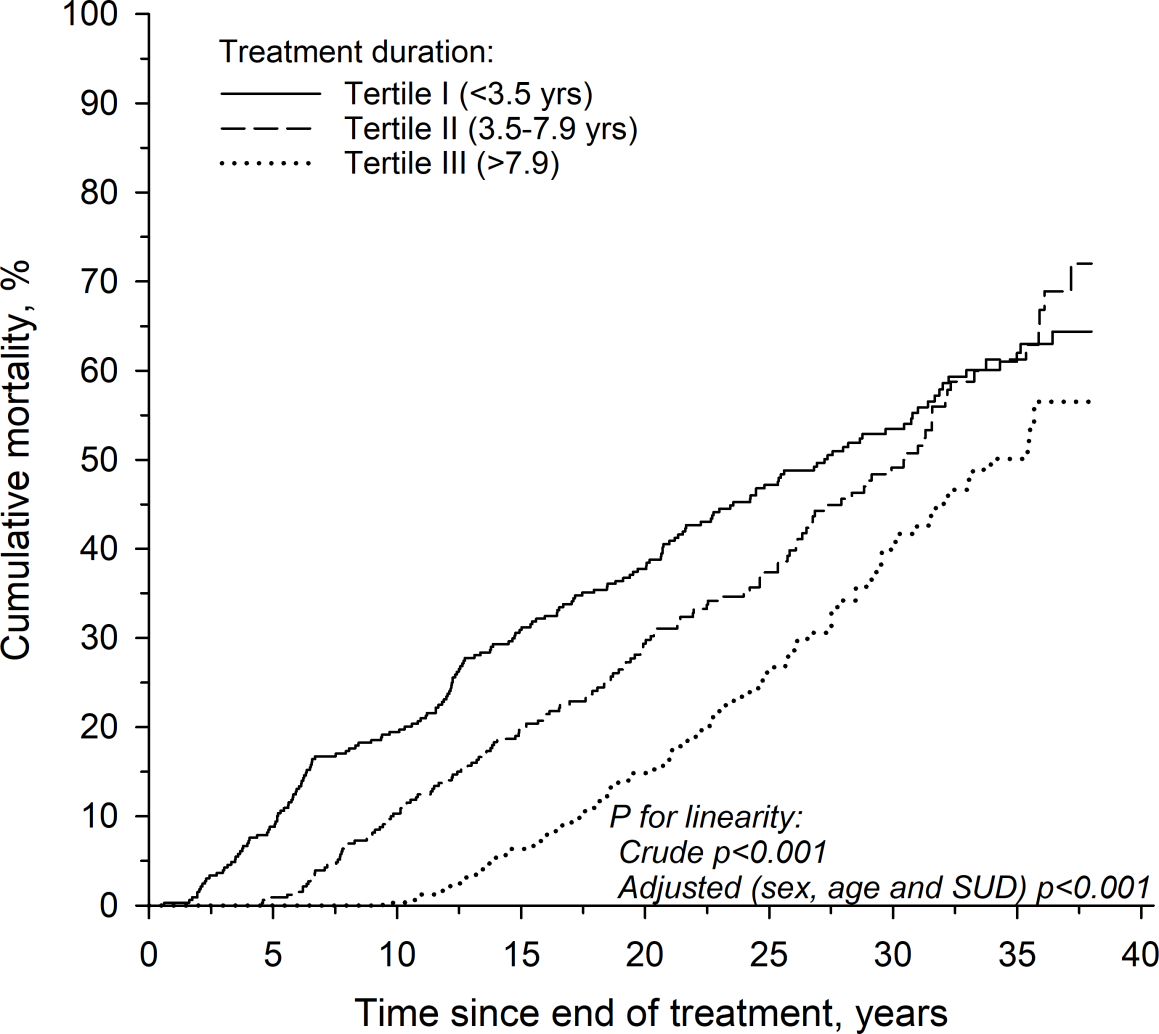
Cumulative (Kaplan-Meier estimate) mortality according to treatment duration tertiles.

**Figure 2:**
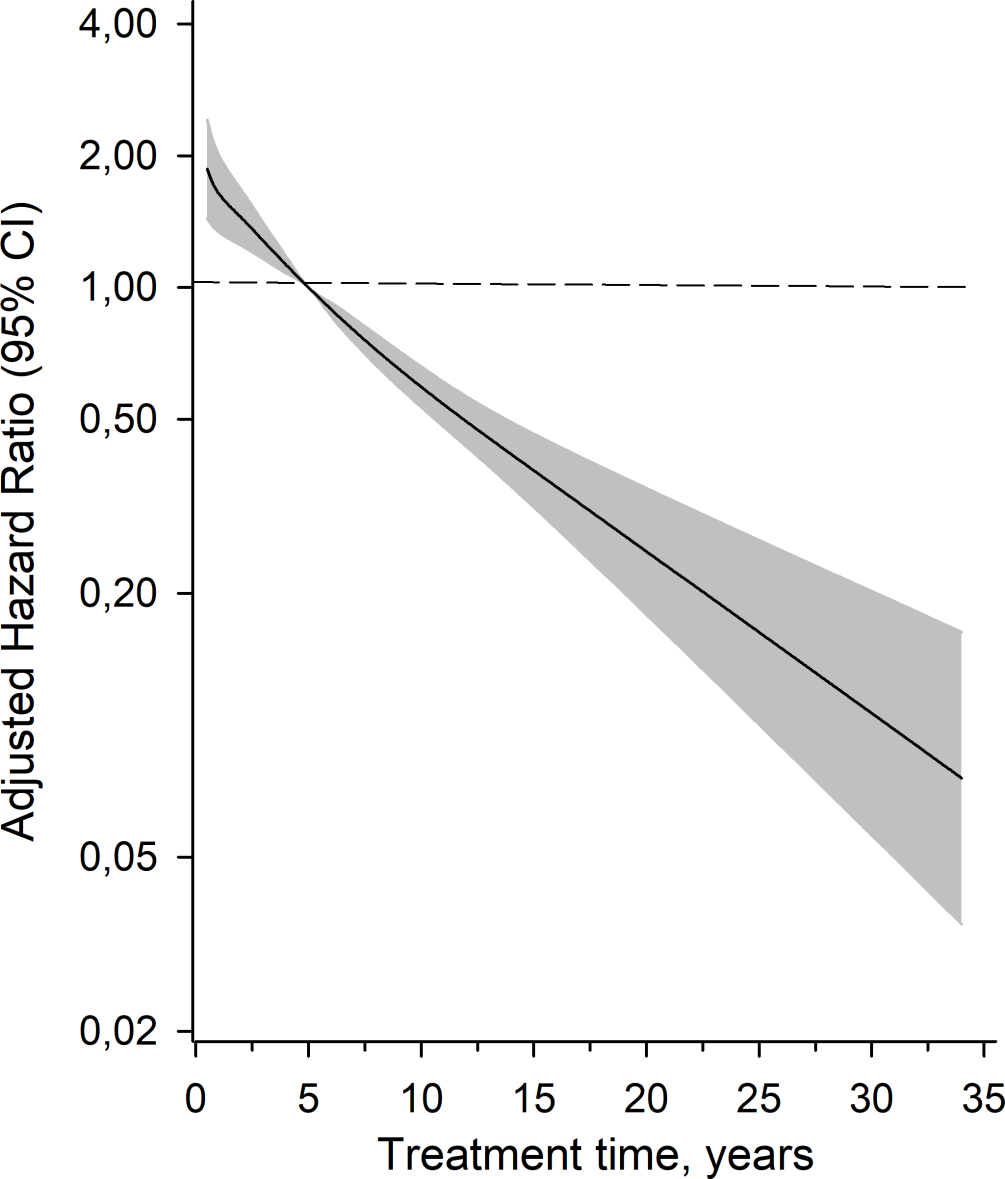
Adjusted hazard ratios for all-cause mortality as a function of the treatment time years calculated model derived from a 3-knot restricted cubic spline Cox proportional hazards regression models. The median time (5 years) was set as the reference. The model was adjusted for age, sex and SUD. Gray area represents the 95% confidence intervals.

The risk associated with treatment duration, age at discharge, gender and presence of SUD to mortality is shown in **Table 2**. The duration of treatment variable was also divided into tertiles. The risk of mortality was highest in the first tertile with the shortest duration of treatment, and lowest in the last tertile (longest duration of treatment). These results are shown in **Table 2**; **Figure 1**. The age and sex adjusted hazard ratios of treatment duration versus mortality for the whole cohort are shown in **Figure 2**. A longer duration of treatment was associated with a lower risk of mortality after release.

**Table 2:** Variables and their association to risk of mortality after treatment in Cox model.

	HR (95% CI)	P-value
Treatment duration (in general)	0.91 (0.89 to 0.92)	<0.001 (Linearity)
I tertile (<3.5 years)	1.00 (Reference)	
II tertile (3.5 - 7.9 years)	0.69 (0.55 to 0.87)	
III tertile (>7.9 years)	0.26 (0.20 to 0.34)	
Age at discharge	1.06 (1.05 to 1.07)	<0.001
Male	1.44 (1.05 to 1.98)	0.024
SUD	1.42 (1.16 to 1.74)	0.001

The data are expressed as mean and standard deviation (SD), or count and percentage (%), as appropriate.

## Discussion

This study estimates the effect of different factors on post-discharge mortality among forensic psychiatric patients in Finland. The results show that a longer duration of treatment is associated with lower risk of mortality. Patients with a SUD are at higher risk of post-discharge mortality. Male sex is also associated with increased post-discharge mortality. Older age at discharge was independently associated with lower post-discharge mortality. In this study, the lower risk of mortality associated to duration of treatment did not seem to abate, indicating that patients may benefit from very long periods of treatment with regard to the risk of mortality.

The results suggest a protective effect of treatment duration on post-discharge mortality in Forensic psychiatric patients suffering from psychotic disorders. There may be several explanations for these findings. Forensic hospitals in Finland provide high quality somatic care in addition to psychiatric care, somatic illnesses are discovered earlier and adequate treatment is initiated sooner compared to patients outside hospitals. Thus, the longer patients stay in hospitals, the more likely will potential chronic illness manifest during this hospital care, rather than outside of it, with proper treatment being initiated in a more timely fashion.

A previous study conducted by our group has shown that the effect of treatment time on recidivism was especially strong for those with a SUD (19). Patients with a SUD may benefit from longer treatment times when forensic psychiatric care focuses also in treating the SUDs. Longer abstinence from substance abuse, provided by a longer during of treatment, may itself also reduce cravings and the risk of SUD relapse after discharge.

Our study, combined with the results of previous studies, suggests that forensic psychiatric patients may receive several benefits from longer treatment times. However, these benefits need to be weighed with the possible negative effects longer treatment may cause, such as getting accustomed to life in facilities, reduction of functionality and reduced return to working force, not to mention loss of self-determination during the hospitalization period (20). Indeed, the issue of self-determination versus prolongation of life merits a nuanced and multifaceted discussion in itself. However, we maintain that well-implemented psychiatric hospitalization–whether involuntary or not and whether forensic or not- should be seen as an example of a necessary and legitimate treatment intervention, particularly when supported by data concerning increased life expectancy. We argue that this reasoning is also in line with the UN’s Istanbul Protocol, which outlines the following: “All health professionals are morally bound by the ethical standards set by their professional bodies and may be judged guilty of professional misconduct if they deviate from professional standards without reasonable justification.” (21). Attempting to increase a patient’s life expectancy should, in our opinion, be seen to fall within the scope of such ethical professional standards, including those of forensic psychiatry, even in an overall context of a psychiatric service striving primarily towards increased self-determination and deinstitutionalization. What is more, it must be noted that in Finland the legal criteria for involuntary psychiatric treatment for both forensic and non-forensic patients are identical, and include the endangering of the person’s own health or safety (Mental Health Act, ch. 2, section 8).

In conclusion and in regards to mortality, longer treatment, especially for male forensic patients with SUDs, seems to reduce the risk of their mortality. However, elongated treatment times risk medical paternalism and must be balanced against patients’ rights to freedom, whilst also recognizing the constitutional right to life. As mortality is one of the most important outcomes, the factors mediating possible decreases in the risks associated with treatment time need to be further studied in order to determine whether they could be used to mitigate risk without elongating in-hospital treatment unnecessarily.

### Strengths and weaknesses

Our study population is nationwide, inclusive, and spans several decades. However, we have only sampled Finnish Forensic Psychiatric Patients and our results may thus not be translatable or generalizable to other countries, especially with different inclusion criteria for forensic patients or differing healthcare and judicial systems. There was no data on comorbid illnesses of the patients, treatment provided to their somatic and mental disorders and either on their living conditions or outpatient care after the discharge from the forensic psychiatric care. Lack of these variables may have an impact to the results and is a weakness of the study. Our study does not include patients who were not released from care during the follow-up period, or died during in-hospital care. Thus, patients spending most of their lifespans in the hospital were not included. Several other skewing factors may also be present. Often, individuals with a milder illness may be released from care earlier than those with a more severe illness. Thus, longer treatment may be related to more severe illness, which may somewhat skew the results presented here. Consequently, the risk reduction associated with longer treatment may be even more pronounced than the severity of illness could account for.

## Data Availability

The datasets presented in this article are not readily and are only available by permission granted by National Institute for Health and Welfare (THL) and by Statistics Finland. Requests to access the datasets should be directed to https://thl.fi/en/web/thl/statistics-and-data/data-and-services/research-use-and-data-permits and to https://www.stat.fi/meta/tietosuoja/kayttolupa_en.html.
